# Quality of life in patients with acromegaly: a scoping review

**DOI:** 10.1186/s13023-024-03246-2

**Published:** 2024-07-04

**Authors:** Wei Wang, Ting Yang, Qinghua Huang

**Affiliations:** Nursing Department, The Second Affiliated Hospital Zhejiang University School of Medicine, Zhejiang, China

**Keywords:** Acromegaly, Quality of life, Psychosocial factors, Scoping review

## Abstract

**Purpose:**

To evaluate the available evidence regarding the quality of life (QoL) in patients with acromegaly, by synthesizing the psychosocial factors of QoL, QoL measures, and complementary interventions targeting QoL.

**Methods:**

A scoping review was conducted using the PRISMA-ScR guideline. We searched six English databases (PubMed, Embase, CINAHL, Scopus, Web of Science, and the Cochrane Library) from the inception to August 21, 2023. We included observational studies involving psychosocial factors and complementary interventions targeting QoL (concept) in patients with acromegaly (population) in any setting (context). The design characteristics, psychosocial factors, measures, details of interventions, and outcomes of included studies were described in detail.

**Results:**

Twenty-one studies were identified, including sixteen cross-sectional studies and five interventional studies. Ten categories of psychosocial factors that are associated with QoL in acromegaly. Depression and anxiety were the most frequent psychosocial factors. Seven different validated QoL measures were used. AcroQoL was the most common measure. Two categories of complementary interventions targeting QoL were identified including psychological and exercise interventions.

**Conclusions:**

Our scoping review provides a reasonably clear picture of the current research status of QoL in acromegaly. However, this review also highlights the need to deepen understanding of QoL and psychosocial factors in the future, as well as conduct longitudinal research and qualitative research to clarify the changing trends of psychosocial factors and specific experiences of patients. Further, more potential clinical complementary interventions are needed to improve QoL for patients with acromegaly.

## Introduction

Acromegaly is a rare and insidious hormonal disorder, characterized by excessive secretion of growth hormone (GH) and insulin-like growth factor-1 (IGF-1) [1]. The prevalence is between 28 and 137 cases per million and the incidence lies between 2 and 11 cases per million per year [2]. As a result of the excessive secretion of GH and IGF-1, patients with acromegaly may experience changes in facial appearance and enlargement of the hands and feet, as well as other multiple system disorders, including cardiovascular disorders, endocrine and metabolic disorders, musculoskeletal disorders, neuropsychological diseases, and malignant neoplasm [3]. A multimodal therapeutic approach can be achieved including surgery, radiation, and medication therapy in clinical practice, which effectively improves the morbidity and mortality of patients [4]. Traditional clinical treatment goals for acromegaly include biochemical control, tumor size reduction, and signs/symptoms improvement [5]. While there is an inconsistency between the biochemical control and the result of quality of life (QoL) [6, 7]. Wolters et al. [6] published a prospective study about the QoL before and during the first 2.5 years of acromegaly treatment. They found all patients had achieved disease control through surgery and/or medication treatment, but QoL remained impaired and was still lower than the general population during the study period. In the other prospective study, Kyriakakis et al. [7] stated that impaired QoL primarily consisted of physical function and psychosocial well-being.

In addition, the diagnosis of acromegaly is often delayed, and some irreversible acromegaly-associated comorbidities may develop during the delay [8]. This means that a significant portion of patients are still affected by the irreversible long-term effects of acromegaly, resulting in impaired QoL [9]. QoL as an important parameter in clinical practice can help healthcare professionals better understand patients’ subjective feelings about the impact of their diseases. The World Health Organization (WHO) defines QoL as “an individual’s perception of their position in life, in the context of culture and value system in their life and relation to their goals, expectations, standards, and concerns” [10]. The goals of health outcomes include reduction of mortality, morbidity and improvement of QoL [11]. However, studies of the impact on the QoL of patients with acromegaly typically focus on biological elements, such as biochemical control and therapeutic approach [12–14]. Comparatively fewer studies explore the psychosocial aspects of QoL, which could provide key information about the impact on QoL of patients with acromegaly.

Psychosocial factors are defined as any exposure that may affect physical health outcomes through psychological mechanisms, including individual cognition, emotion, personality, social interaction, and so on [15], which are candidate modifiable factors to QoL for patients with acromegaly. Acromegaly has been reported to be associated with psychological comorbidities such as depression [16] and anxiety [17]. A recent study showed the superiority of psychopathology (depression and anxiety) over biochemical control and other variables in predicting QoL [18]. Further, acromegaly is associated with progressive morphometric changes. It has been reported that negative perception of body image is related to impaired social relationships QoL domain in other pituitary tumor patients [19]. Thus, the assessment and therapy of psychosocial factors for patients with acromegaly need to be considered.

It is necessary to raise again that biochemical control does not mean an improvement of QoL. So the complementary interventions targeting QoL beyond conventional therapy (surgery, radiation, and medication therapy) become of paramount importance. Yet, the existing literature on interventions targeting QoL is heterogeneous and inconclusive. In 2017, Geraedts et al. [20] conducted a systematic review of predictors of QoL in patients with acromegaly. They found depressive symptoms and BMI were significant predictors of QoL in acromegaly, and only interventions with lanreotide autogel and pegvisomant were shown to consistently improve QoL. Broersen et al. [21] reported QoL improved during acromegaly treatment in a systematic review, most of the studies they included identified medication as the main treatment.

Therefore, in this review, using the framework of Arksey and O’Malley [22], we aimed to comprehensively review psychosocial factors of QoL, QoL measures, and complementary interventions target QoL, with the specific aims of describing the designs and the defining features of included studies. The specific research questions that guided this scoping review were as follows: (1) What psychosocial factors affect the QoL in patients with acromegaly? (2) What types of complementary interventions target QoL beyond conventional therapy (surgery, radiation, and medication therapy) in patients with acromegaly? 3)What are measures for QoL in patients with acromegaly?

## Methods

This scoping review is conducted following the framework of Arksey and O’Malley and the Preferred Reporting Items for Systematic Reviews and Meta-Analyses extension for Scoping Reviews (PRISMA-ScR) [23]. PRISMA-ScR checklist is presented in Appendix 1. This scoping review was not registered.

### Search strategy

Our research team consulted 2 information specialists and reviewed previous relevant studies to develop search strategies. We performed a comprehensive literature search using six electronic databases, including PubMed, Embase, CINAHL, Scopus, Web of Science, and the Cochrane Library. All databases were searched from the inception to August 21, 2023. The reference lists in the included studies were traced back to identify additional studies. The search strategy combined terms for (1) acromegaly, and (2) QoL, and has been included as Appendix 2.

### Eligibility criteria

The eligibility criteria were developed according to the PCC (Participants /Concept /Context) structure [24]. Participants were diagnosed with acromegaly by any available diagnostic criteria. The concept of this scoping review was observational studies involving psychosocial factors (e.g. cross-sectional studies or cohort studies) and interventions targeting QoL in patients with acromegaly. And in our scoping review, there were no limits to the context.

We excluded interventions that target QoL through medical therapy (e.g. pegvisomant, lanreotide, somatostatin), and the interventions compared different treatment methods (e.g. surgery vs. medicine). QoL measures that have not been validated (e.g. Patient Assessed Acromegaly Symptom Questionnaire, PASQ) and didn’t focus on overall QoL were also excluded. In addition, newspaper articles, comments, and conference abstracts were not included.

### Study selection

EndNote X9 was used to manage all the retrieved studies. The study selection was conducted in two steps. In the first step, two investigators independently reviewed the titles and abstracts against the inclusion and exclusion criteria. The full text of potentially relevant studies was screened against the eligibility criteria in the second step. Any disagreements were resolved by consensus with a third review investigator.

### Data extraction and charting

A standardized data extraction table was developed by our research team. The relevant data on psychosocial factors were extracted, including author, year of publication, country, study design, sample, disease duration, psychosocial measure, and QoL measure. The relevant data of interventions target QoL in patients with acromegaly were extracted, including author, year of publication, country, study design, sample, type of intervention, content of intervention, and does. Two investigators independently extracted data according to the eligibility criteria. In case of disagreements, a third investigator was involved.

## Results

### Overview of selected papers

**Fig. 1:**
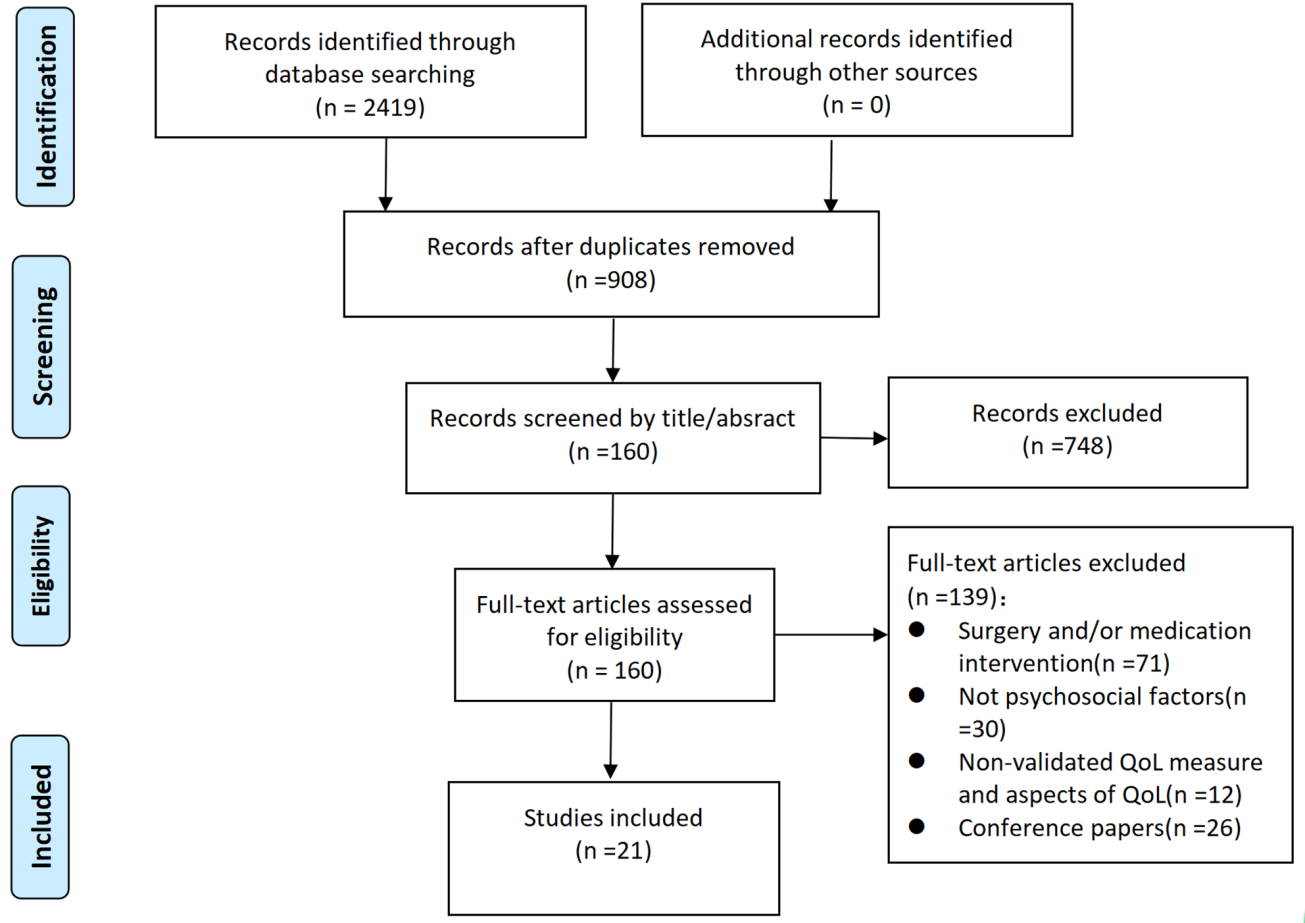
Flow chart of study selection process

The six electronic databases and references screening yielded 2419 studies. We removed 1511 duplicates, leaving 908 studies. Of these studies, 748 were excluded through the title and abstract screening process, and 160 studies were subsequently full-text reviewed. 139 studies were excluded with reasons: surgery and/or medication interventions (*n* = 71); not psychosocial factors (*n* = 30); non-validated QoL measure and aspects of QoL (*n* = 12); conference papers (*n* = 26). We ultimately included 21 studies [18, 25–44] in our review, including sixteen cross-sectional studies [18, 25–39], and five intervention studies [40–44]. A flow chart of the study selection is presented in Fig. 1.

### Design characteristics

Sixteen cross-sectional studies were published between 2004 and 2022 [18, 25–39], and the sample size ranged from 40 [32] to 291 [36] patients. Four studies were conducted in the Netherlands [30, 34, 35, 38], three in Italy [25, 28, 39], two in China [33, 37] and Turkey [26, 27] respectively, and a single study in Belgium [36], Germany [18], Greece [32], Mexico [29] and Poland [31] respectively.

Five intervention studies were published between 2014 and 2023 [40–44], including one randomized controlled trial [40], two non-randomized clinical trials [42, 44], and two self-comparison before and after trials [41, 43]. The sample size ranged from 7 [41] to 45 [40] patients. Two studies were conducted in Brazil [42, 43] and Turkey [41, 44] respectively, and one multicenter study [40] in Spain and Italy. Tables 1 and 2 show an overview of the studies included.

**Table 1:** Psychosocial factors of QoL in patients with acromegaly.

Author (year)	Country	Design	Sample	Age(years)	Sex(M/F)	Disease duration (years)	Therapy	Psychosocial measure	Quality of Life measure
**Depression**									
Cangiano (2022)	Italy	cross-sectional	n = 171	20–85	76/95	**-**	Surgery(n = 90) Medication (n = 157) Radiotherapy (n = 26)	Depression AIMS	AcroQoL
Geraedts (2015)	Germany	cross-sectional	n = 80	54.7 ± 12.3	37/43	10.8 ± 10.0	**-**	BDI	AcroQoL and SF−36
Kepicoglu (2014)	Turkey	cross-sectional	n = 133	47 ± 11.5	52/81	8.1 ± 6.9	Surgery(n = 114) Medication (n = 133) Radiotherapy (n = 38)	BDI	AcroQoL
Celik(2013)	Turkey	cross-sectional	n = 57	**-**	0/57	4(2.25,13)	Surgery(n = 45) Medication (n = 40) Radiotherapy (n = 15)	BDI	AcroQoL
Pivonello (2022)	Italy	cross-sectional	n = 223	56(48,64.5)	94/129	5(2,10)	**-**	BDI-II	AcroQoL
Ballesteros (2021)	Mexico	cross-sectional	n = 85	38.0 ± 13.6	38/47	-	**-**	BDI	AcroQoL and SF−36
**Anxiety**									
Geraedts (2015)	Germany	cross-sectional	n = 80	54.7 ± 12.3	37/43	10.8 ± 10.0		STAI	AcroQoL and SF−36
Pivonello (2022)	Italy	cross-sectional	n = 223	56(48,64.5)	94/129	5(2,10)		STAI Form Y 1 and 2	AcroQoL
Ballesteros (2021)	Mexico	cross-sectional	n = 85	38.0 ± 13.6	38/47	-	**-**	BAI	AcroQoL and SF−36
**Depression and anxiety**									
Biermasz (2004)	the Netherlands	cross-sectional	n = 118	58.6 ± 12.9	61/57	**-**	**-**	HADS	AcroQoL, SF−36 and NHP
**Psychiatric distress**									
Jawiarczyk (2020)	Poland	cross-sectional	n = 50	51.7 ± 14.5	19/31	8.4 ± 8.8	**-**	GHQ	AcroQoL and WHOQoL-BREF
Anagnostis (2014)	Greece		n = 40	60 ± 2.0	15/25	-	Surgery(n = 31) Medication (n = 23) Radiotherapy (n = 13)	POMS	AcroQoL
***Body image***									
Zhang (2022)	China	cross-sectional	n = 68	46.4 ± 12.5	31/37	**-**		BICI	SF−36
Roerink (2015)	the Netherlands	cross-sectional	n = 73	59.4 ± 10.5	40/33	**-**	Surgery(n = 64) Medication (n = 39) Radiotherapy (n = 14)	DAS59	AcroQoL and RAND−36
***Illness perceptions***									
Tiemensma (2011)	the Netherlands	cross-sectional	n = 81	60 ± 12	47/34	**-**		IPQ-R	AcroQoL, EuroQoL−5D and Physical Symptoms Checklist
T'Sjoen (2007)	Belgium	cross-sectional	n = 291	54.8 ± 13.5	149/142	**-**	-	SSS	AcroQoL
***Acceptance of illness***									
Jawiarczyk (2020)	Poland	cross-sectional	n = 50	51.7 ± 14.5	19/31	8.4 ± 8.8	**-**	AIS	AcroQoL and WHOQoL-BREF
***Stigma***									
Li(2022)	China	cross-sectional	n = 102	45.3 ± 13.5	42/60	**-**	Surgery(n = 89) Medication (n = 13)	SSCI	AcroQoL
***Medicine beliefs***									
Andela (2015)	the Netherlands	cross-sectional	n = 73	60.1 ± 11.6	40/33	16.1 ± 10.0	Surgery(n = 62) Medication (n = 28) Radiotherapy (n = 17)	BMQ	AcroQoL and EuroQoL−5D
***Sleep quality***									
Wennberg (2019)	Italy	cross-sectional	n = 67	56(48,64.5)	26/41	30 (11, 42.5)	Medication (n = 57) Radiotherapy (n = 6)	PSQI	AcroQoL

**Table:** Continued Table 1

Author (year)	Results
*Depression*	
Cangiano(2022)	Depression was significantly negatively correlated with AcroQoL.
Geraedts (2015)	Depression can be significantly predictive of QoL measured by AcroQoL and SF−36.
Kepicoglu(2014)	Depression was significantly negatively correlated with AcroQoL.
Celik(2013)	A strong negative correlation was found between AcroQoL total score and BDI score.
Pivonello(2022)	BDI-II total score was the best predictor of AcroQoL physical domain score.
Ballesteros(2021)	The higher scores of BDI correlated with lower QoL as assessed by global, and all subdomain scores in AcroQoL.
***Anxiety***	
Geraedts (2015)	Anxiety can be significantly predictive of QoL measured by AcroQoL and SF−36.
Pivonello(2022)	STAI Y 1 and 2 score correlated with AcroQoL total score.
Ballesteros(2021)	The higher scores of BAI correlated with lower QoL as assessed by global, and all subdomain scores in AcroQoL.
***Depression and anxiety***	
Biermasz(2004)	HADS were significantly negatively correlated with AcroQoL, SF−36 and NHP.
***Psychiatric distress***	
Jawiarczyk(2020)	No association between the scores of GHQ−28 and QoL.
Anagnostis(2014)	AcroQoL scores were negatively associated with POMS.
***Body image***	
Zhang(2022)	The BICI score was negatively correlated with SF−36.
Roerink(2015)	DAS59 scores were significantly negatively correlated with AcroQoL.
***Illness perceptions***	
Tiemensma(2011)	Illness perceptions was significantly negatively correlated with AcroQoL, EQ−5D, and Physical Symptoms Checklist.
T'Sjoen(2007)	SSS was significantly negatively correlated with AcroQoL.
***Acceptance of illness***	
Jawiarczyk(2020)	The level of acceptance of illness was significantly positively correlated with AcroQoL and WHOQoL-BREF.
***Stigma***	
Li(2022)	Total stigma was significantly positively correlated with the AcroQoL.
***Medicine beliefs***	
Andela(2015)	BMQ subscale Specific-Necessity SA was negatively associated with AcroQoL subscales psychological-appearance and psychological-personal relations and the total score on the AcroQoL.
***Sleep quality***	
Wennberg(2019)	Sleep quality was associated with poorer overall AcroQoL, physical AcroQoL, psychologicalAcroQoL, and social AcroQoL

**Table 2:** Characteristics of included intervention studies.

Author (year)	Country	Design	Sample	Type of intervention	Content of intervention	Dose
*Psychological interventions*						
Santos (2023)	Spain and Italy	Multicentre randomized controlled trial	n = 45	Mindfulness therapy	Centre 1: Mindfulness-Based Stress Reduction Centre 2: Compassion and Mindfulness Based Daily Life Therapy	Meditate 6 days per week, In Centre 1 formal meditation 45 minutes per day, informal meditation was no specific time request. In Centre 2, both formal and informal meditation 20 minutes per day, and also had other tasks related to daily life activities.
Halilolu (2020)	Turkey	Self comparison before and after	n = 7	Psychological focus group therapy	Using insight-oriented and supportive psychother-apy techniques, encouraging patients to try new techniques of socialization and talk about different aspects of the illness.	Weekly group psychotherapy sessions.
Kunzler(2018)	Brazil	Non-randomized clinical trial	n = 20	Cognitive-behavioral therapy (CBT)	Basic concepts of CBT; emotional regulation; increase self-esteem and self-confidence, and supply of a brain-shaped piggy bank; “Think healthy and feel the difference” technique; the technique for approaching anger, assertiveness training, and the coping card; the technique for approaching fear; the technique adapted for shame about one’s physical appearance in acromegaly; clarifications on acromegaly; a summary of the topics covered in the sessions.	Nine weekly 90-min “Think healthy” group therapy sessions.
***Exercise interventions***						
Lima (2019)	Brazil	Self comparison before and after	n = 17	Therapist-oriented home rehabilitation	5 min of warm-up exercises, 20 min of muscle strengthening and resistance exercises for the upper and lower limbs, 10 min of balance training, 20 min of aerobic training, 5 min of global stretching and relaxation exercises.	Before starting the protocol, the patients were instructed by how to perform the physical exercises, and followed an exercise programme from a booklet with instructions for each exercise prescribed for 3 times a week, for a total of 24 sessions.
Hatipoglu (2014)	Turkey	Non-randomized clinical trial	n = 20	Exercise	Each session consisted of warming up, cardio, strength, balance and stretching. At the end of each month, the difficulty of the exercises was increased.	Exercised 3 days a week for three consecutive months. And each exercise session, which lasted 75 min, was supervised.

**Table:** Continued Table 2

Author (year)	Providers	Control	Duration	Outcomes	Improvement of QoL
Santos (2023)	Mindfulness teacher and research staff	Normal clinical routine	8 weeks	Quality of life, mood, pain, sleep, self-compassion, life satisfaction, blood pressure and heart rate	There was no change in QoL.
Halilolu (2020)	Psychoanalyst and clinical psychologist	-	12 months	Quality of life and depression	There was an effective improvment on QoL.
Kunzler (2018)	-	-	3 months	Quality of life and depression	There was an effective improvment on QoL.
Lima (2019)	Physiotherapist	-	2 months	Quality of life, fatigue, body composition, handgrip strength, lower extremity functionality, body balance and functional capacity	QoL was improved during the intervention period, while the gain was lost after 1 month of washout.
Hatipoglu (2014)	-	-	3 months	Quality of life, BMI, hormone levels, depression and body image.	There was no change in QoL.

#### Psychosocial factors of QoL in patients with acromegaly

The eligible studies reported on ten categories of psychosocial factors of QoL. The most prevalent psychosocial factors in the included studies were depression (*n* = 7) [18, 25–30], and anxiety (*n* = 4) [18, 28–30]. Two studies assessed psychiatric distress [31, 32], body image [33, 34] and illness perceptions [35, 36] respectively. There were single studies for acceptance of illness [31], stigma [37], and medicine beliefs [38] and sleep quality [39].

#### The QoL measures

A total of 7 validated QoL measures were used in our scoping review, including the Acromegaly Quality of Life Questionnaire (AcroQoL), Short Form 36-item Health Survey (SF-36), EuroQol Five Dimensions Questionnaire (EuroQoL-5D), WHO Quality of Life Scale-BREF (WHOQoL-BREF), Nottingham Health Profile (NHP), RAND-36 and Physical Symptoms Checklist. SF-36 and RAND-36 include the same items, but the recommended scoring algorithm is somewhat different. Two studies used three QoL measures as an outcome [30, 35], and five studies used two QoL measures [18, 29, 31, 34, 38]. The AcroQoL was the most frequently used QoL measure in 15 studies [18, 25–32, 34–39]. SF-36 was used to assess in 4 studies [18, 29, 30, 33], and EuroQoL-5D in 2 studies [35, 38].

#### The influence of the psychosocial factors of QoL in patients with acromegaly

In the studies we included involving depression, the general conclusion was that depression was significantly negatively correlated with QoL [18, 25–29]. Studies involving anxiety found that a high level of anxiety was associated with a worse QoL [18, 28, 29]. Two studies found that psychiatric distress was associated with a lower QoL [31, 32]. Two studies that investigated body image found that higher scores of body image were associated with a lower QoL [33, 34]. Illness perceptions were found to be associated with QoL negatively in 2 studies [35, 36]. One study found that greater acceptance of illness was more likely to have a higher QoL [31]. Higher stigma was significantly associated with a lower QoL [37]. One study that investigated medicine beliefs found that stronger beliefs about specific necessity to stay healthy are related to a worse QoL [38]. A poorer sleep quality was reported that associated with a worse QoL [39].

#### Interventions target QoL in patients with acromegaly

Two categories of interventions targeting QoL were identified including psychological (*n* = 3) [40–42] and exercise (*n* = 2) [43, 44] interventions. Psychological interventions included mindfulness therapy, psychological focus group therapy, and cognitive-behavioral therapy. Exercise interventions included therapist-oriented home rehabilitation and exercise.

Santos et al. [40] conducted an 8-week mindfulness therapy in patients with acromegaly that evaluated the effect on quality of life. However, there was no change in QoL. Halilolu et al. [41] designed a self-comparison before and after trial using psychological focus group therapy and found it was a useful intervention positively affecting QoL and depression. Kunzler et al. [42] developed a cognitive-behavioral therapy technique“Think Healthy”, showing an effective improvement in the quality of life.

Lima et al. [43] designed a 2-month therapist-oriented home rehabilitation therapy and found the QoL was improved during the intervention period, while the gain was lost after 1 month of washout. Hatipoglu et al. [44] used a non-randomized clinical trial to explore the impact of exercise programs on QoL in patients with acromegaly. After 3 months, the score of AcroQoL was not changed.

## Discussion

This scoping review highlights psychosocial factors of QoL and measures used to assess the QoL in patients with acromegaly, as well as nonpharmacological and/or surgical interventions targeting QoL. 21 studies were synthesized across 10 countries from 2004 to 2023, of which the details were described in our review.

As stated by the WHO, there are three patient-related health outcomes in chronic disease management: reducing mortality, reducing morbidity, and improving QoL [45]. As the life expectancy of patients with acromegaly increases, more and more researchers believe that QoL should be managed as an independent treatment goal in acromegaly [46]. This is the first scoping review of QoL in patients with acromegaly, which provides a clear mapping for the development of QoL in patients with acromegaly.

Successful surgery or medication can improve the quality of life of patients, but it is rarely completely normalized and not always associated with biochemical markers.

We only included 5 interventions targeting QoL. In the preliminary screening, we found that there were a large number of surgery, medication, and radiation intervention studies whose outcomes included QoL, which was the challenge we encountered whether such studies should be included. For patients with well-defined acromegaly, surgery, medication, and radiation therapy were common methods in clinical practice. Successful surgery or medical treatment could improve the patient’s QoL, but it is rarely completely normalized and not always associated with biochemical markers [47]. Medical treatment, could lower GH and IGF-1, and improve both comorbidities of acromegaly and QoL. However, the long-term need for monthly injections of somatostatin analogs to control the disease may hurt AcroQoL scores [48]. In recent decades, the life expectancy of patients with acromegaly has also significantly increased, requiring new complementary methods that can alleviate physical and emotional consequences and the long-term disease burden on the health system [49]. So we only included interventions targeting QoL beyond these conventional methods. In our review, only psychological focus group therapy and cognitive-behavioral therapy showed potential in QoL by Halilolu et al. [41] and Kunzler et al. [42], and Kunzler et al. [50] conducted a 9-month follow-up at the end of the cognitive-behavioral intervention, they found the effects of intervention were maintained. However, the intervention benefits of therapist-oriented home rehabilitation carried out by Lima et al. [43] disappeared after one month of washout, so the long-term effect of intervention is an aspect that researchers need to consider when designing. While the findings are not conclusive as only a small number of interventions were included, it provides a new perspective on designing interventions to improve QoL. There are all small sample sizes in the included interventions, which is an unavoidable difficulty in rare diseases such acromegaly. Further work with large randomized controlled trials and strong experimental designs are needed to replicate these benefits and more potential clinical complementary interventions are needed to improve QoL for patients with acromegaly.

Ten categories of psychosocial factors that are associated with QoL in acromegaly include depression, anxiety, psychiatric distress, body image, illness perceptions, acceptance of illness, cognitive function, stigma, medicine beliefs, and sleep quality. Most psychosocial factors are modifiable and might provide valuable targets for future interventions. Depression and anxiety are the most frequent psychosocial factors investigated. A cross-sectional survey shows that the prevalence of psychiatric comorbidity in patients with acromegaly ranges from 40 to 50% [17]. Due to the disruption of body image, patients with acromegaly are more likely to exhibit anxiety-related personality traits [51]. Arthropathy is associated with poor mood and QoL [52, 53]. Cangiano et al. reported that 28% of patients with acromegaly displayed depression. The proportion was significantly higher than those reported in subjects with primary osteoarthritis of the hand and in patients without osteoarthritis [25]. Arthropathy is a common and disabling complication of acromegaly, and it does not seem to entirely regress after hormonal normalization, so therapies are often unable to restore joint function to its previous state. Almost three out of four patients complain of articular pain, stiffness, or limitations during both active and biochemically controlled disease [53]. Moreover, Tseng et al. reported that presence of comorbidities might affect QoL of patients with acromegaly, and patients with diabetes mellitus (DM) had lower psychological score and psychological scores than those without DM [54]. A recent study emphasizes that the significant decrease in the QoL of patients with acromegaly is mainly driven by psychopathology rather than biochemical control of the disease. It is recommended to conduct systematic psychopathological screening and specific psychological treatment for acromegaly to improve the QoL of patients [18]. In our review, the included studies were cross-sectional designs that evaluated the impact of psychosocial factors on QoL at the current time point. We cannot know how psychosocial factors affect QoL over time. The cross-sectional design excludes some possible influencing factors in modulating QoL in patients affected with acromegaly. In addition, the lack of qualitative research also makes it difficult for us to understand the true thoughts of patients with acromegaly. Further work with longitudinal research and qualitative research should be conducted to clarify the changing trends of psychosocial factors and specific experiences of patients.

There were 7 different QoL measures utilized across the 16 studies. Most studies use specific QoL measures of acromegaly, the Acromegaly Quality of Life Questionnaire (AcroQol). AcroQol is the most widely used tool for measuring the QoL in acromegaly currently, the effectiveness and clinical applicability have been validated in a 6-month prospective study [55]. Standardized measures can increase comparability between different studies. However, there is a discrepancy in cultural background and medical level among different countries, so researchers should test or develop QoL measures based on the treatment and psychosocial characteristics of patients with acromegaly in their own country. In addition, we also call on researchers to use the COSMIN risk of bias checklist [56] as a guide to comprehensively and objectively evaluate the performance of the developed tool.

### Limitations

Though we proposed a strict screening and search strategy among the six major databases to determine a widespread belief in results, there were still some limitations that need to be addressed. First, the lack of qualitative and mixed methods of research endangers a deeper understanding of the characteristics that affect QoL, as trends, specific experiences, and insights can only be captured through these methods. Second, in our scoping review, we only included studies that focus on overall QoL and QoL measurement tools must be validated, which may result in missing relevant studies. Third, considering the breadth of the definition of ‘psychosocial factors’, there may also be a range of other factors that have not yet been reported. Finally, we only searched the English database, which may lead to publication bias due to the omission of other language literature.

## Conclusions

Our scoping review identified ten categories of psychosocial factors for QoL in acromegaly, seven validated QoL measures, and two categories of interventions targeting QoL, which provide a reasonably clear picture of the current research status of QoL in acromegaly. However, this review also highlights the need to deepen understanding of QoL and psychosocial factors in the future, as well as conduct longitudinal research and qualitative research to clarify the changing trends of psychosocial factors and specific experiences of patients. Further, more potential clinical complementary interventions are needed to improve QoL for patients with acromegaly.

## Data Availability

All data generated or analysed during this stage are included in this published article [and its Additional files].
